# Comparison between the two drug regimens of PPI+Amoxicillin+ Rifampicin and PPI+Amoxicillin+ Levofloxacin for the treatment of *H. pylori* infections resistant to the first line drug regimen among patients referred to Ilam clinics

**Published:** 2019

**Authors:** Ghobad Abangah, Alireza Raughani, Parisa Asadollahi, Khairollah Asadollahi

**Affiliations:** 1 *Department of Internal Medicine, Faculty of Medicine, Ilam University of Medical Sciences, Ilam, Iran*; 2 *Faculty of Medicine, Ilam University of Medical Sciences, Ilam, Iran*; 3 *Department of Microbiology, School of Medicine, Iran University of Medical Sciences, Tehran, Iran*; 4 *Department of Microbiology, Faculty of Medicine, Ilam University of Medical Sciences, Ilam, Iran*; 5 *Department of Social Medicine, Faculty of Medicine, Ilam University of Medical Sciences, Ilam, Iran *

**Keywords:** *Helicobacter pylori*, Levofloxacin, Rifampicin, PPI, Drug resistance

## Abstract

**Aim:**

This study compares the effects of two drug regimens for *Helicobacter pylori* (*H. pylori*) infection resistant to the first line drug regimen among patients referred to Ilam clinics, Iran.

**Background:**

Single drug regimen is not effective for *H. pylori* infection and therefore, application of triple or quadruple drug regimens are currently applied.

**Methods:**

This study was performed by a before-after comparative method and patients were randomly selected among those consecutively referred to Ilam gastrointestinal clinics. Patients with failure in the first line treatment, were randomly divided into two equal groups and each group was treated by one of the PPI+Amoxicillin+Rifampicin or PPI+Amoxicillin+Levofloxacin drug regimens for 14 days. Six weeks after treatment, patients were tested for *H. pylori* stool antigen and the results were compared between two groups.

**Results:**

In this study, 100 patients including 49 (49%) men and 51 (51%) women were examined. There was no statistical difference between the two groups for gender, age and living location at the start of study (p = 0.068). The mean age of the patients was 44.55 ±15.1 years old ranging from 17 to 85 years. Response to treatment among the levofloxacin group, was 90% and in rifampicin group 72% with a significant difference (p<0.04).

**Conclusion:**

The response rate of *H. pylori* infection to the Levofloxacin based regimen was 90%; however, the application of rifampicin in combination with other drugs against *H. pylori* infection (72% response rate), should be limited to reduce the possibility of drug resistance in case of tuberculosis infection.

## Introduction

 Helicobacter pylori (*H. pylori*) is a gram-negative flagellated bacillus that naturally colonizes the human's gastric mucosa which can persist the acidic medium of stomach and infect human for their entire lifetime (1-3). Without an effective treatment, this organism could rarely be eliminated and relapses after treatment (4). The prevalence rate of this infection, increasing with age is about 30% among developed countries and up to 80% among developing countries and the prevalence rate among individuals above 35 years old in Iran has been reported as 90% (5-9). Even though most studies have rejected any association between *H. pylori* prevalence and gender, some have reported a 10% higher prevalence among males compared to females which believed might be associated with more antibiotic consumption among females (5-6, 9-10). 

Selection of a suitable method for the eradication of *H. pylori* infection depends on the type and price of drugs, accessibility, side effects, facility of use, patients' conditions and drug resistance and whether the method is invasive or non- invasive (6-11). Chronic gastric inflammation could be the most important consequence of H. pylori infection that could result in active chronic gastritis or duodenal ulcer. There is a potent association between peptic ulcer and gastric cancer, and considering this consequence, an accurate and quick diagnosis and appropriate therapy of this organism is vital (12). 

Different antibiotics have been applied by different regimens for this infection in different areas (13). The last trials indicated that when the first line drug therapies have failed, the regimen including levofloxacin and amoxicillin accompanied by a proton pump inhibitor (PPI) for 14 days is the best option for the eradication of *H. pylori* infection. Comparison between levofloxacin based drug regimens with other quadruple drug regimens has confirmed its high effectiveness; however, there are concerns about H. pylori resistance against this useful drug and further studies are necessary to be carried out before its wide application (14).

 Triple drug regimen, with an eradication rate of 50%, has been introduced as the first line treatment (15). The goal of therapy is an eradication rate of about 85 -90%; however, since the success of two drug therapy is less than 80%, such a regimen is not suggested. Application of triple drug regimens has some limitations including lack of patients' cooperation and side effects but patients' cooperation could be achieved by simplification of therapy such as consumption of drugs twice daily (16). 

A study by Kuo and colleagues (2009) reported that levofloxacin based regimen was more effective in eradication of the H. pylori infection than a quadruple regimen without this drug and levofloxacin based regimen was emphasized by the researchers (17). Another study by Romano (2010) reported an eradication rate of 80.8% and 96.8% for two regimens based on clarithromycin and levofloxacin, respectively but their difference was not significant (18). Similar studies have compared different regimens in various conditions and most of their reports emphasized on levofloxacin based regimen in eradication of *H. pylori* infection (19-23). 

The effectiveness of triple or quadruple drug regimen based on metronidazole is applied in Iran; however, due to high resistance to metronidazole in our country, the eradication rate of H. pylori infection by regimen based on metronidazole is not considerable (24, 26-27). Therefore, different regimens in Iran have been applied by researchers and mostly emphasized on higher efficacy of quadruple drug regimens based on clarithromycin or furazolidone (27-30). Due to common complications of furazolidone and high price of clarithromycin some alternative drug regimen for treatment of H. pylori infection are needed.

Rifampicin is one of the drugs used for treatment of *H. pylori* infection by clinicians and due to its better tolerance and twice daily application as well as its lower duration therapy (10-14 days) is preferred. In addition, rifampicin is applied in triple drug regimens and it is preferred and tolerated by patients in comparison with quadruple drugs regimens. Therefore, the current study aimed to compare the two triple drug regimens of PPI+Amoxicillin+Rifampicin and PPI+Amoxicillin+Levofloxacin for the treatment of *H. pylori* infection among patients with a response failure against the first line drug therapy. 

## Methods

This study was performed by a before-after comparative method. The samples for this study were randomly selected among patients referred to Ilam gastrointestinal clinics during 2016. Patients with continued gastrointestinal (GI) symptoms and a response failure to the first line drug therapy, who underwent endoscopy procedure and their pathologic and experimental tests confirmed *H. pylori* infection were participated in this study.

After completion of an informed consent form, patients were randomly divided into two groups. One group was treated by PPI+Amoxicillin+Rifampicin drug regimen and another group by PPI+Amoxicillin+Levofloxacin for two weeks. Four weeks after treatment duration, patients were visited again and were tested for *H. pylori* stool antigen. Excluding criteria included patients who could not complete the treatment duration. 

The drug regimen for both groups included a proton pump inhibitor such as omeperazol 20mg twice daily and 1 gram amoxicillin twice daily; however, as the third drug 250 mg levofloxacin twice daily was applied for levofloxacin group and 150 mg rifampicin twice daily for rifampicin group and the duration of therapy was considered as 14 days for both groups. Patients were advised to consume their drugs on time and complete their therapy duration and report any side effect as soon as possible. At the end of each week, patients were called to report any complaint they might have. Four weeks after treatment, patients were revisited to report any signs and symptoms. Finally, a H. pylori stool antigen test was obtained from each patient. A negative stool antigen test and lack of any GI complaint by patient was considered as H. pylori eradication. Data associated with each patient including gender, age, clinical symptoms, drug side effects and H. pylori stool antigen results were entered into prepared forms. All patients' data were entered into SPSS 20 and based on the type and numbers of variables comparison between the two groups was carried out using t test and chi squared tests. 

This study was approved by Ethics Committee of Ilam University of Medical Sciences. 

## Results

All participants including 100 patients completed their therapy and their associated data was analyzed. In the levofloxacin group, 26 males (52%) and 24 females (48%) and in the rifampicin group, 23 males (46%) and 27 females (54%) were analyzed and the difference between the two groups for gender was not significant (p=0.68). The mean age of the patients was 44.55± 15.1 years old ranging from 17 to 85 years. Seventy percent of patients in the levofloxacin and 78% of those in the rifampicin groups were from urban areas without any significant difference (p=0.49). Demographic data of the participants is indicated in table 1. 

There was no significant difference between two groups for demographic data at the start of study.

The most frequent GI symptoms among either levofloxacin or rifampicin groups was gastric pain with 58% and 66%, respectively without any significant difference between the two groups (p=0.74).

After treatment, the elimination rate of all symptoms was 92% for the levofloxacin group and 82% for the rifampicin group. Even though the symptom elimination rate in the levofloxacin group was higher and more patients were symptom free, the difference between the two groups was not significant (p=0.23).

**Table 1:** Demographic data of patients with *H. pylori* infection resistant to the first line treatment

Variable		Drug regimen group		P
		Levofloxacin N (%)	Rifampicin N (%)	
Gender	Male	26 (52)	23 (46)	0.68
	Female	24 (48)	27 (54)	
Place of life	Urban	35 (70)	39 (78)	0.49
	Rural	15 (30)	11 (22)	
Age (year)	<40	22(49)	26 (54)	0.06
	40-60	24 (63)	14 (37)	
	>60	4 (29)	10 (71)	

**Table 2:** Comparison between the frequencies of GI symptoms among patients with *H. pylori* infection resistant to the first line treatment before the study

GI symptom	Drug regimen group		P
	Levofloxacin N (%)	Rifampicin N (%)	
			0.74
Gastric pain	29 (58)	33 (66)	
GERD (Reflux)	10 (20)	10 (20)	
Left upper quadrant pain	2 (4)	0 (0)	
GI bleeding	2 (4)	1 (2)	
Nausea	5 (10)	4(8)	
Dysphasia	2 (4)	2 (4)	
Total	50 (100)	50 (100)	

**Table 3:** Comparison between the elimination rate of GI symptoms among patients with H. pylori infection resistant to the first line treatment after the study

GI symptom	Drug regimen group		P
	Levofloxacin N (%)	Rifampicin N (%)	
			0.74
No	46 (92)	41 (82)	
Yes	4 (8)	9 (18)	
Total	50 (100)	50 (100)	

**Table 4:** Comparison between the results of H. pylori stool antigen test among patients with response failure to the first line treatment at the end of study

Fecal H. pylori antigen test	Drug regimen group		P
	Levofloxacin N (%)	Rifampicin N (%)	
			0.04
Positive	5 (10)	14 (28)	
Negative	45 (90)	36 (72)	
Total	50 (100)	50 (100)	

Four weeks after the treatments, the results of *H. pylori* stool antigen test showed 90% elimination rate for the levofloxacin group and 72% for the rifampicin group, with a significant difference between the two groups (p=0.04).

The eradication rate of *H. pylori* infection based on stool antigen test for both drug regimens was almost in accordance with patients symptomless and was more considerable in drug regimen based on levofloxacin. 

The eradication rate of *H. pylori* infection per protocol of omeperazol 20mg twice daily + 1 gr amoxicillin twice daily + 250mg levofloxacin twice daily for two weeks was 90% and per protocol of omeperazol 20mg twice daily + 1 gr amoxicillin twice daily+ 150 mg rifampicin twice daily for two weeks was 72%.

## Discussion

Antibiotic resistance in *H. pylori* infection is increasing and the main objective of the current study was to compare and determine the effects of two triple drug regimens among patients infected by *H. pylori *resistant to the first line treatment in Ilam city, Iran.

Up to now, different drug regimens have been attempted for *H. pylori *eradication in different countries including Iran; however, clinical experiences in developing countries including in Iran indicated that the eradication rate using uniform drug regimens, was much less than that in developed countries (5-9). In a study from Brazil, the resistance rate of this microorganism to metronidazole, amoxicillin, clarithromycin and tetracycline has been reported as 42%, 29%, 7% and 7%, respectively (31). In addition, the relapse rate either in short or long-term duration was much higher than that reported in developed countries (32).

According to the results of present study, the eradication rate among those who received levofloxacin regimen was higher (90%) compared to those who received rifampicin regimen (72%). The most frequently symptom among both groups was gastric pain that was eradicated by 92% among levofloxacin regimen group and by 82% among rifampicin regimen group at the end of study; however, the difference was not significant. 

In a prospective study by Vander Poorten and others (2007), the effectiveness of a triple drug regimen based on rifabutin was investigated among 67 patients and an eradication rate of 76% was reported whilst 24% of the patients still had complains about symptoms associated with H. pylori infection (33). The elimination rate in the current study was higher than that of Poorten study, which might be related to the difference in methods, sample sizes or in the employed drugs in drug regimens. 

The method, duration and kinds of used drugs in any drug regimen, are among affecting factors in the outcome and effectiveness of treatment in patients with *H. pylori* infection. The results of current study showed that based on stool Ag test, the eradication rate of patients treated by regimen including levofloxacin was 90% but 72% by the regimen including rifampicin with a significant difference. Though patients selected for the current study were among those with failure in response to the first line treatment; however, their responses to the applied regimens, particularly in combination with levofloxacin, was better than the previous reports even among those without failure responses in the first line treatment (34-35). In a study by Mantzari and others from Greece, the eradication rate of H. pylori infection by a triple drug regimen including omeprazole, amoxicillin and metronidazole was 65% (36). The eradication rate of *H. pylori* infection in another study by Catalano and colleagues from Germany during triple and quadruple drug regimens was 76% and 85%, respectively (37) which still were lower than the regimen applied in the current study based on levofloxacin. A similar study by Kuo and colleagues (2009) in Taiwan compared two drug regimens among patients with failure in response to the standard *H. pylori* treatment. In their study, the effects of a triple drug regimen including levofloxacin, amoxicillin and esomeprazole was compared to a quadruple drug regimen including bismuth subcitrate, tetracycline, metronidazole and esomeprazole. They reported a similar effect for both regimens but levofloxacin based regimen was more effective in eradication of the infection and was emphasized by the researchers (17). The method applied in Kuo study was almost similar to the current study particularly in selecting patients with failure to the first line therapy as well as in the regimen including levofloxacin; however, the drug regimens compared in each study were different. Both studies confirmed a higher effect for the levofloxacin based regimen; however, in contrast to the Kuo study, the difference between the two compared regimens was significant in the current study which might be associated with a lower number of the drugs used in the current study (quadruple regimen in Kuo and triple regimen in the current study). 

In a study by Francesco and colleagues from Italy, patients with H. pylori induced peptic ulcer underwent medical treatment for 10-days duration. At the first 5 days, patients received 20mg omeprazole twice daily plus one gram amoxicillin twice daily, and at the second five days they received 20mg omeprazole twice daily, 500mg clarithromycin twice daily and 500mg tinidazole twice daily. The eradication rate for H. pylori infection in their study was reported as 96.8%, which shows a highly effective regimen in comparison with other applied drug regimens (38). Despite the high effectiveness of their method in a lower duration, their regimen was only slightly more effective than that applied in our study (90%); however, the patients participated in Francesco study did not have the criteria of response failure to the first line drugs. In addition, the number of applied drugs in their regimen was more, compared to that in the current study which is most likely accompanied by higher expenditure and drug side effects. 

Another study by Romano and others (2010) from Italy, reported an eradication rate of 80.8% and 96.8% for two regimens based on clarithromycin and levofloxacin respectively but their difference was not significant (18). Despite the difference in the drug combination and the selected patients between Romano and our study, both studies confirmed a better effectiveness of the levofloxacin based drug regimen. Gisbert *et al.* (2013) compared a triple drug regimen based on levofloxacin and a quadruple drug regimen for 10 days and reported a higher eradication rate for triple drug regimen including levofloxacin (20).

In a study by Basu and colleagues from the USA (2011), the eradication rates of 10 and 7-days treatment by levofloxacin based regimen was reported as 90% and 88.9%, respectively and that for 10-days treatment by clarithromycin based regimen as 73.3%. Basu and colleagues concluded that the regimens including levofloxacin, even in 7-days duration of therapy, indicated a better eradication rate for *H. pylori* infection (19). The eradication rate obtained in the current study was the same as Basu study for levofloxacin and 72% for rifampicin that was in accordance with the rate reported for clarithromycin by Basu study. However, there were two differences between these two studies; the duration of therapy in the current study was longer (14 days) and the patients selected were among those with failure in the first line treatment. 

Another study by Gisbert *et al*. from Spain was performed on 1000 patients who had failure in the first line treatment. They compared a triple drug regimen including 500mg levofloxacin twice daily, 1 gram amoxicillin twice daily and 20mg omeprazole twice daily for 10 days with a regimen including bismuth instead of levofloxacin. The eradication rates reported by the authors for levofloxacin based regimen were 76%, 68%, 70%, 76%, 74% and 81% in 2006, 2007, 2008, 2009, 2010 and 2011, respectively and concluded that the regimen including levofloxacin had higher effects compared to the regimen including bismuth (20). Based on their reports in the different years, the maximum eradication rate for levofloxacin was 81% that was lower than that reported in the current study (90%). Considering the similar drug types, dosages and participants (resistance in the first line therapy), this difference could be associated with the duration of therapy that was 10 days in their study compared to 14 days in the current study. It seems that two-week treatment with levofloxacin based regimen, reveals a better eradication rate compared to the 10-day or lower duration for patients with failure in the first line treatment. 

Application of rifampicin, one of the main effective antibiotics for the treatment of tuberculosis, is controversial against *H. pylori* infection either in regard with its dosage or duration of therapy. 

On the other hand, there are concerns about the possibility of drug resistance in case of tuberculosis infection if rifampicin is widely and improperly used for other purposes. Studies by Cianci and colleagues (2006) and Cammarota and others (2004), revealed that the application of rifampicin, amoxicillin and PPI, which was similar to the regimen used in the current study, had a high effectiveness in eradication of *H. pylori* infection (39-40). In addition, Sanaka (1999), Pilotto (2000), Fujimura (2002) and their colleagues also reported a high effectiveness rate of the regimens involving rifampicin in the treatment of *H. pylori *infections (41-43). On the other hand, a study by Ahuja and colleagues (2005), reported that rifampicin in combination with tetracycline and esomeprazole, was ineffective against *H. pylori* infection (44). 

According to the results of the present study and many other reports, the 2-weeks application of levofloxacin based drug regimens reduces the rate of *H. pylori* infection among patients with response failure against the first line treatment by 90%. However, the application of rifampicin in combination with other drugs against *H. pylori* infection (with a response rate of 72%), should be limited to reduce the possibility of drug resistance in case of tuberculosis infection. The effectiveness of the regimen will decrease by reducing the treatment duration less than 14 days.
